# The Interconnection between Hepatic Insulin Resistance and Metabolic Dysfunction-Associated Steatotic Liver Disease—The Transition from an Adipocentric to Liver-Centric Approach

**DOI:** 10.3390/cimb45110570

**Published:** 2023-11-14

**Authors:** Milena Vesković, Nikola Šutulović, Dragan Hrnčić, Olivera Stanojlović, Djuro Macut, Dušan Mladenović

**Affiliations:** 1 Institute of Pathophysiology “Ljubodrag Buba Mihailovic”, Faculty of Medicine, University of Belgrade, 11000 Belgrade, Serbia; milena.veskovic@med.bg.ac.rs; 2 Institute of Medical Physiology “Richard Burian”, Faculty of Medicine, University of Belgrade, 11000 Belgrade, Serbia; nikolasutulovic@gmail.com (N.Š.); drhrncic@yahoo.com (D.H.); ostanoj@gmail.com (O.S.); 3 Clinic of Endocrinology, Diabetes and Metabolic Diseases, Faculty of Medicine, University of Belgrade, 11000 Belgrade, Serbia; djmacut@gmail.com

**Keywords:** insulin resistance, NAFLD, hepatokines, ER stress, circadian clock, low-grade inflammation, adipose tissue, lipotoxicity

## Abstract

The central mechanism involved in the pathogenesis of MAFLD is insulin resistance with hyperinsulinemia, which stimulates triglyceride synthesis and accumulation in the liver. On the other side, triglyceride and free fatty acid accumulation in hepatocytes promotes insulin resistance via oxidative stress, endoplasmic reticulum stress, lipotoxicity, and the increased secretion of hepatokines. Cytokines and adipokines cause insulin resistance, thus promoting lipolysis in adipose tissue and ectopic fat deposition in the muscles and liver. Free fatty acids along with cytokines and adipokines contribute to insulin resistance in the liver via the activation of numerous signaling pathways. The secretion of hepatokines, hormone-like proteins, primarily by hepatocytes is disturbed and impairs signaling pathways, causing metabolic dysregulation in the liver. ER stress and unfolded protein response play significant roles in insulin resistance aggravation through the activation of apoptosis, inflammatory response, and insulin signaling impairment mediated via IRE1/PERK/ATF6 signaling pathways and the upregulation of SREBP 1c. Circadian rhythm derangement and biological clock desynchronization are related to metabolic disorders, insulin resistance, and NAFLD, suggesting clock genes as a potential target for new therapeutic strategies. This review aims to summarize the mechanisms of hepatic insulin resistance involved in NAFLD development and progression.

## 1. Introduction

Metabolic homeostasis represents the balance between the intake and requirements for nutrients. Multi-organ cooperation is achieved through neural and humoral regulatory mechanisms that convey information about the physiological state of the organs, their needs, and the flow of nutrients so that they can be adequately used or stored [1].

One of the most important hormones for the regulation of metabolic homeostasis is insulin, which represents an anabolic peptide hormone and is secreted by pancreatic β cells. Even though the major role of insulin is the regulation of glucose homeostasis, there is a spectrum of various additional roles of this hormone [2]. Numerous pathways are activated or inhibited by the action of insulin through its receptor. It has been shown that insulin increases glucose uptake, glycogen synthesis, lipogenesis, protein synthesis, gene expression, DNA synthesis, the uptake of amino acids, and the activity of the Na^+^/K^+^ pump. On the other hand, insulin has the ability to inhibit gluconeogenesis, lipolysis, apoptosis, and autophagy [2,3].

The liver represents a dynamic organ responsible for metabolic regulation and homeostasis. Hepatic functions are numerous and include lipid and cholesterol metabolism, macronutrient metabolism, support of the immune system, glucose homeostasis, and blood volume regulation and also play a detoxification role through the breakdown of xenobiotics and drugs [4]. There are numerous endogenous and exogenous factors that can affect liver function and lead to various diseases. One of the most common liver diseases is non-alcoholic fatty liver disease (NAFLD) [5].

NAFLD is defined as the accumulation of fat in hepatocytes, predominantly in the form of triglycerides, where at least 5% of hepatocytes must contain lipid droplets in the absence of excessive alcohol intake [6]. It is often asymptomatic in the early phase of the disease and can be present for years and go undetected. Worldwide, NAFLD is considered to be a global health problem that has rapidly increasing prevalence, especially among the young population [7]. Parallel with NAFLD, the prevalence of diabetes mellitus type 2 (T2DM) is rising significantly, accompanied by obesity, which is increasingly prevalent in children [8]. The global prevalence of NAFLD is 30% and increasing, requiring serious and urgent comprehensive strategies for a better understanding of the course of the disease and to raise awareness at all levels of society and the scientific community [7]. Metabolic comorbidities associated with NAFLD include obesity, T2DM, hyperlipidemia, hypertension, and metabolic syndrome (MetS) [9]. All of these comorbidities are known as crucial risk factors for cardiovascular diseases, which are the leading causes of death in NAFLD patients [10,11].

**Figure 1:**
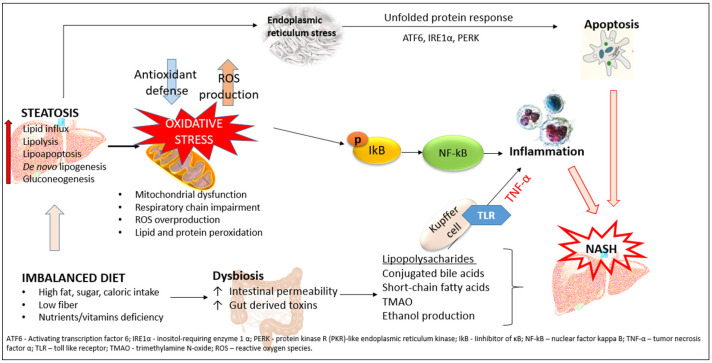
Pathogenic mechanisms involved in steatosis progression to NASH. Accumulating fatty acids causes respiratory chain disruption, mitochondrial impairment, and reactive oxygen species (ROS) overproduction, suggesting that oxidative stress is the main event in NASH development. ROS and lipid peroxidation activate the endoplasmic reticulum stress response and cause apoptosis in the case of inadequate unfolded protein response. ROS induce inflammation in an NF-κB-dependent manner with subsequent release of cytokines and chemokines. An additional factor that contributes to NASH development is dysbiosis and increased intestinal permeability. This leads to increased release of lipopolysaccharides and other metabolites into the portal circulation, which activates Kupffer cells and promotes inflammation and NASH.

NAFLD is usually defined as a hepatic manifestation of MetS and represents a wide spectrum of liver diseases, including simple steatosis, non-alcoholic steatohepatitis (NASH), and liver fibrosis with the potential to progress into cirrhosis and hepatocellular carcinoma (HCC) [5,12]. However, recent studies emphasize the bidirectional link between NAFLD and metabolic syndrome. The central mechanism involved in the pathogenesis of NAFLD is insulin resistance with hyperinsulinemia which stimulates triglyceride synthesis in the liver [13]. On the other side, triglyceride and free fatty acid accumulation in hepatocytes promotes insulin resistance via oxidative stress, endoplasmic reticulum (ER) stress, lipotoxicity, and the increased secretion of hepatokines [14]. These additional factors are responsible for NAFLD progression into NASH, and they are the reason for replacing the two-hit theory with the multiple-hit theory in NASH pathogenesis (Figure 1).

Due to severe metabolic impairment caused by altered glucose and lipid metabolism, NAFLD is now called metabolic-dysfunction-associated steatotic liver disease (MASLD). This encompasses individuals with a fatty liver but who also have at least one cardiometabolic risk factor [15]. The interplay between MASLD, obesity, and insulin resistance is widely accepted, confirming numerous complex interactions among the liver and other endocrine axes. Strong evidence suggests that endocrine dysregulation has direct and indirect effects on the development and severity of liver disease [16]. One widely accepted theory explaining the development of MASLD primarily involves insulin resistance. Obesity, closely linked to insulin resistance, significantly increases the risk of MASLD, with approximately 30–90% of obese individuals developing hepatic steatosis [17]. The bidirectional relation between insulin resistance and MASLD represents one of the reasons for the still insufficiently efficient therapy of these systemic metabolic disturbances associated with fatty liver disease. Based on this background. This review aims to summarize the data from the currently available literature on the link between insulin resistance and MASLD with emphasis on the liver-centric role in the aggravation of insulin resistance and NASH development.

## 2. The Role of the Circadian Clock in Insulin Resistance and MASLD Development

Numerous metabolic diseases have been associated with disturbances of circadian rhythm and the biological clock. The circadian regulatory system consists of a central clock in the suprachiasmatic nucleus (SCN) of the anterior hypothalamus and multiple peripheral clocks in other brain areas and peripheral organs and tissues [18]. The major role of the central clock is to synchronize peripheral clocks, but there are also specific roles in sleep–wake behavior, food intake, hormone secretion, and insulin sensitivity which can all affect glucose metabolism [19,20]. Central clock genes that regulate the biological function of circadian rhythm are period genes (PER1, PER2, and PER3), cryptochrome genes (CRY 1 and CRY2), BMAL1, CLOCK, and genes encoding nuclear receptor REV-ERB and ROR [20]. Misalignment between central and peripheral clocks seems to be the major underlying explanation for the association between circadian misalignment and impaired insulin signaling [21]. A recent study by Jouffe et al. [22] suggested that disruption of BMAL1 activity significantly impacts the pathogenesis of metabolic and liver diseases. BMAL1 knockout mice fed a high-fat diet were prone to obesity but did not develop insulin resistance. Mice lacking CRY1, CRY2, or liver-specific BMAL1 or REV-ERB genes were prone to obesity and gained weight rapidly with metabolic disturbances when fed a high-fat diet [23]. However, time-restricted feeding in these animals protected the mice from excessive weight gain, lipid accumulation in hepatocytes, hyperlipidemia, and metabolic changes, suggesting that imposed feeding–fasting rhythms can override compromised rhythms and metabolic oscillations [23]. Differentiated embryonic chondrocyte gene 1 (DEC1) modulates circadian phases of some clock genes, and in patients with obstructive sleep apnea DEC1 and BMAL1, they were upregulated, while PER1 was reduced. These parameters were associated with MASLD components such as hyperlipidemia and obesity and correlated with insulin resistance, oxidative indicators, and inflammatory IL-6 [24]. The metabolic state reflecting glucose and lipid metabolism changes differently in the morning and in the evening and is strongly associated with the circadian clock [25]. Further and deeper research needs to be performed in order to find new approaches for ameliorating metabolic disorders by targeting biological clock and circadian synchronization. Recent research conducted in vitro on cultured hepatocytes showed that BMAL1 and CLOCK play an important role in the regulation of hepatic lipid homeostasis by modulating the SREBP1c/PPARγ signaling pathway [26], confirming the significant role of circadian rhythm in MASLD development and progression [27,28,29].

## 3. The Liver-Centric Role in Insulin Resistance

**Figure 2:**
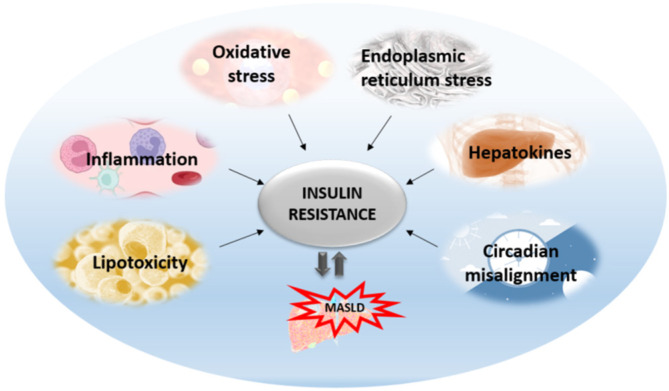
Mechanisms affecting hepatic insulin resistance and contributing to its aggravation and MASLD progression.

Insulin resistance is the pathophysiological hallmark of MASLD. Previously, it was thought that MASLD was only a consequence of insulin resistance. Today, we know that MASLD aggravates insulin resistance and the relationship between these two entities is bidirectional. The most accepted is the liver-centric approach suggesting that MASLD affects extra-hepatic organs as well as causing metabolic disturbances, with the major pathogenic mechanisms originating from the liver. The most significant mechanisms that are activated as lipids start to accumulate in the liver include oxidative stress, ER stress, inflammation, apoptosis, altered autophagy, and the effects of hepatokines (Figure 2). All these hepatic mechanisms are described further in more detail.

### 3.1. Hepatic ER Stress and Unfolded Protein Response in Insulin Resistance

**Figure 3:**
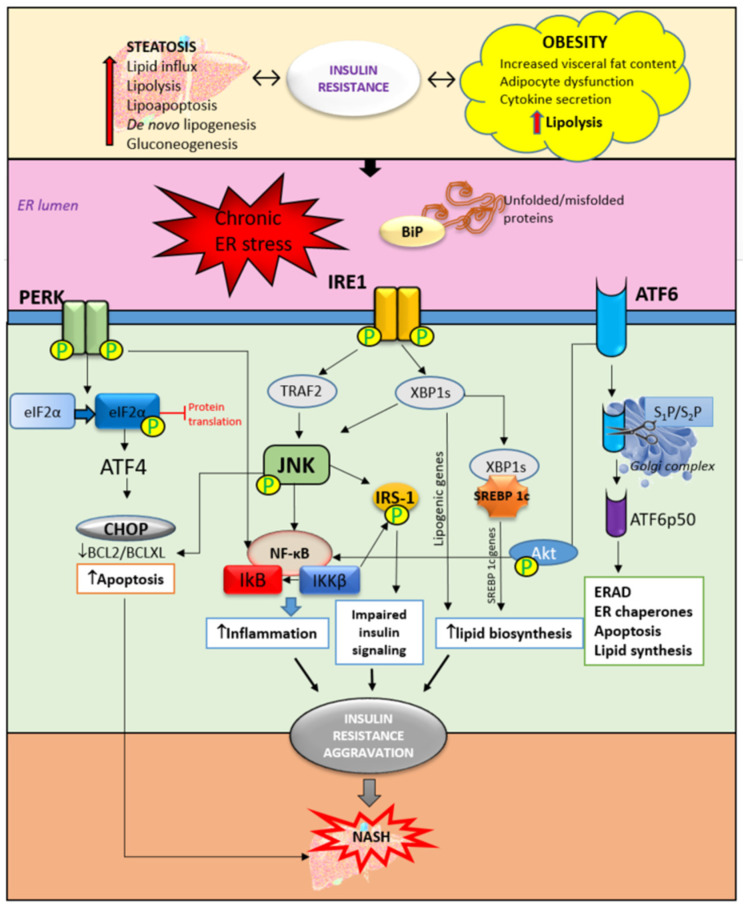
The role of ER stress and UPR activation in insulin resistance and NASH development. Obesity and liver steatosis are bidirectionally connected with insulin resistance and are known to induce ER stress and consequently UPR activation. UPR is mediated through three pathways. The accumulation of unfolded and/or misfolded proteins trigger the activation of UPR. The activation and phosphorylation of IRE1, PERK, and ATF6 trigger an inflammatory response through JNK/NF-κB activation. Furthermore, impaired insulin signaling is mediated by the phosphorylation of IRS-1 mediated by JNK activation. In addition, ER stress triggers upregulation of the SREBP 1c receptor which contributes to lipid biosynthesis and accumulation. Accompanied by increased apoptosis of hepatocytes, these mechanisms are responsible, at least partly, for the insulin resistance aggravation contributing to NASH development. ER—endoplasmic reticulum, BiP—immunoglobulin heavy chain-binding protein, IRE1—inositol-requiring enzyme, PERK—PKR-like ER kinase, ATF6—activating transcription factor 6, eIF2α—eukaryotic initiation factor-2α, CHOP—C/EBP homologous protein, XBP1—XBP1, X-box binding protein 1, TRAF2—TNF receptor-associated factor 2, JNK—c-jun-N-terminal kinase, IRS1—insulin receptor substrate-1, ERAD—ER-associated degradation, SREBP—sterol regulatory element binding protein, IκB—inhibitor of nuclear factor kappa B, NF-κB—nuclear factor kappa B, and IKKβ—inhibitor of nuclear factor kappa-B kinase subunit beta.

The ER is a cell organelle, continuous membrane structure with multiple functions, particularly important for protein synthesis, folding, modification and transport, calcium homeostasis, and lipid biogenesis [30]. Recently, various studies have shown that dysfunction of the ER plays an important role in insulin resistance and, subsequently, MASLD through the activation of ER stress signaling [31,32] (Figure 3). Studies on animal models of MASLD, as well as in obese patients, have confirmed the link between ER stress and obesity by the presence of ER stress markers in the steatotic liver [33,34]. In addition, weight loss and a significant reduction in body mass were correlated with the improvement and lowering of ER stress markers [35]. During stress conditions, misfolded or unfolded proteins accumulate in the ER, triggering the unfolded protein response (UPR). The UPR is activated in order to restore normal cell function through the suspension of further protein translation through the degradation of misfolded proteins and the production of more chaperones that are responsible for protein folding. Three types of ER-resident stress sensors are activated as a part of the UPR. They include inositol-requiring enzyme (IRE1), PKR-like ER kinase (PERK), and activating transcription factor 6 (ATF6) [36,37]. In the liver, UPR protects hepatocytes from cellular stress, exogenous toxins, and chemicals. It has been shown that UPR is involved in the regulation of lipid homeostasis in hepatocytes, suggesting that chronic, prolonged ER stress may play an important role in MASLD pathogenesis by affecting lipid metabolism in hepatocytes through altered VLDL secretion [38,39], inducing de novo lipogenesis [40], and impairing insulin signaling and autophagy [41,42]. In addition, the accumulation of lipids in hepatocytes can trigger ER stress, thus proposing a bidirectional relationship between steatosis and ER stress. On the contrary, there is a one study on mice lacking hepatic IRE1 that showed increased steatosis and even profound NASH development after 20 weeks on a high-fat diet [43,44]. Moreover, inhibition of the PERK/AFT4/CHOP signaling pathway with celastrol treatment protected mouse hepatocytes and prevented the progression of MASLD induced by a high-fat diet [41]. A similar study showed that downregulation of CHOP gene expression ameliorated ER stress in hepatocytes in high-fat diet-induced MASLD in rats [44]. The study by Nasiri-Ansari et al. showed that UPR pathways PERK, IRE1, and ATF6 were downregulated in the liver tissue in empagliflozin-treated animals with MASLD [33]. This pathway is involved in the regulation of lipogenesis and steatosis, as shown in ATF-4-deficient mice in which decreased synthesis of fatty acids and decreased serum triglycerides were noticed [42,45]. Furthermore, ER stress and autophagy are also correlated, since autophagy is activated under ER stress conditions; so-called ER stress-mediated autophagy includes the degradation of protein aggregates, damaged organelles, and misfolded proteins. A new branch of macroautophagy is ER-phagy in which the autophagosome membrane selectively includes parts of the ER membrane [43,46,47,48].

IRE1 has its kinase function which is responsible for c-Jun-N-terminal kinase (JNK) and inhibitory kappa B (IκB) kinase phosphorylation. Together with ER stress-activated lipogenesis, the JNK signaling pathway contributes to insulin resistance development in the liver. JNK and IκB are involved in triggering inflammatory responses and pro-apoptotic pathways [43,44]. In NASH, the inflammatory response is predominantly mediated by the activation of nuclear factor kappa-light-chain-enhancer of activated B cells (NF-κB) contributing to steatohepatitis progression into severe forms of liver injury such as cirrhosis, fibrosis, and HCC [48]. In hepatocytes, ER stress-activated PERK reduces the translation of IκB and subsequently increases the activity of NF-κB. Additionally, ATF6 can also potentiate NF-κB activation through Akt phosphorylation, further promoting inflammation in the liver. There is a strong relationship between JNK-dependent hepatocyte injury and the activation of NF-κB in Kupffer cells releasing proinflammatory cytokines such as IL-1, IL-6, and TNF-α. Besides the promotion of hepatic inflammation, ER stress and UPR can lead to the interruption of insulin signaling through inhibition of the maturation of insulin proreceptors, affecting the transportation of newly synthesized insulin proreceptors from the ER to the cell membrane in vitro [49]. The results from the same study reported that insulin-stimulated Akt phosphorylation was inhibited after 8 to 12 h of ER stress independently of JNK. Instead, reduced Akt phosphorylation was accompanied by the depletion of β-chains of mature insulin receptors and the accumulation of unprocessed α-β precursors of the insulin receptor in the ER [49]. In recent years, ER stress has increasingly been the focus of metabolic disorder research and, above all, liver diseases. A better understanding of the signaling pathways involved in UPR opens up new possibilities for therapeutic targeting.

### 3.2. Insulin Resistance—From Adipose Tissue to the Liver

Insulin resistance is manifested as the reduced ability of insulin to inhibit glucose production in the liver and to stimulate the utilization of glucose in adipose tissue and skeletal muscles [50]. Insulin resistance is a cardinal feature of MASLD and is more prevalent in patients with NASH compared to those with simple steatosis [51,52]. In the state of insulin resistance, fat tissue lipolysis is increased as well as circulating levels of free fatty acids, increasing their efflux from adipose tissue to the liver. Insulin resistance can be central (hepatic) and peripheral (skeletal muscle and adipose tissue). The peripheral form is manifested as the reduced uptake of glucose from blood in the muscles and fat tissue with increased efflux of free fatty acids, while the central form is manifested as the uncontrolled production of hepatic glucose resulting from the impaired suppression of gluconeogenesis and glycogen synthesis [53,54].

Obesity is defined as abnormal or excessive fat accumulation that causes health issues, including increased risk of cardiovascular diseases [55] as well as malignancy [56]. Visceral adipose tissue is a metabolically active and inflammatory organ influencing both glucose and lipid metabolism that can modulate the metabolic processes and function of the liver, skeletal muscles, brain, and cardiovascular system [57]. Fat accumulation in the visceral adipose tissue causes adipocyte hypoxia, ER stress, and adipokine imbalance which all together promote low-grade inflammation with increased secretion of proinflammatory cytokines (tumor necrosis factor (TNF)-α and interleukin (IL)-6 and IL-1) [51,56]. Cytokines and adipokines cause insulin resistance, thus promoting lipolysis in adipose tissue and ectopic fat deposition in the muscles and liver [58]. Free fatty acids along with cytokines and adipokines contribute to insulin resistance in the liver via the activation of numerous signaling pathways including the inhibitor of κB-kinase-β (Iκκβ), c-Jun N-terminal kinase (JNK), protein kinase C, and protein tyrosine phosphatase 1b (PTP1b). Activation of these signaling pathways contributes to the development of inflammation and fibrogenesis [58,59].

Adipokine secretion, dysfunctional adipose tissue, dyslipidemia, and subsequent systemic low-grade inflammation play a dominant role in the development of fatty liver disease and MASLD [59]. Reduced adiponectin release is one of the most important factors in MASLD development, and its decreased concentration is linked with obesity and increased body fat. Adiponectin has hepatoprotective effects due to its ability to reduce inflammation, inhibiting the release of proinflammatory cytokines such as IL-6 and TNF-α [60,61] and improving insulin resistance. Adiponectin also reduces the influx of free fatty acids (FFAs) in the liver and prevents steatosis development through 5–AMP kinase inhibiting acetyl-CoA decarboxylase (ACC) and fatty synthase [62,63]. Recently, adiponectin was shown to be a strong stimulatory factor for maintaining ATP-linked respiration in cultured β-cells. Oxidative phosphorylation and glucose-dependent insulin secretion are restored by the presence of adiponectin for both the insulin-secreting INS1 cell line and primary islets [64]. Adding adiponectin to cells treated with plasma from obese donors significantly restored β-cell function, indicating that a lack of this hormone causes dysfunction of and damage to β-cells [64].

Besides adiponectin, leptin, the hormone responsible for food intake regulation, also plays an important role in obesity. In contrast to adiponectin, the leptin concentration increases in obesity and insulin resistance due to increased adipose tissue mass. Leptin is secreted by adipocytes and carried by the bloodstream to the hypothalamus, with it sending information to the brain about the stored fat amount. Probably due to dysfunction of the leptin receptor, the body develops leptin resistance, promoting steatosis and insulin resistance in patients with prediabetes, with or without MASLD [65]. Leptin has been shown to exert proinflammatory and profibrotic effects in MASLD through the upregulation of macrophages, neutrophils, IL-6, and TNF-α [66,67]. Dysfunctional adipose tissue also contributes to MASLD pathogenesis through the delivery of fats and adipokines to the liver, leading to steatosis and liver inflammation [68].

Crosstalk between adipose tissue and the liver is a key mechanism underlying MASLD development and progression. The major source of non-esterified fatty acids (NEFAs) is peripheral fat stored in adipose tissue [69]. After release from fat depots, NEFAs flow to the liver and accumulate in the form of triglycerides. Furthermore, dysfunctional adipose tissue downregulates the expression of glucose transporter 4 (GLUT4) in adipocytes, causing dysregulation of glucose metabolism and insulin resistance in the liver [69,70]. Increased expression of GLUT4 in mice, improves glucose tolerance and insulin sensitivity. On the other hand, in mice deficient in GLUT4 transporter, even with normal adiposity, insulin resistance and whole-body glucose intolerance were observed [70]. During insulin resistance, stored triglycerides undergo a higher rate of breakdown that increase releasing of the FFA into circulation. Circulating FFAs activate the proinflammatory NF-κB pathway in the liver, resulting in lipotoxicity [71].

Lipotoxicity contributes to MASLD development in combination with triglycerides, biliary acids, free cholesterol, ceramides, and lysophosphatidyl cholines [72]. Predominantly, fat accumulates in the liver in the form of triglycerides, which are derived from glycerol esterification and FFAs. Sources of free fatty acids are dietary intake, lipolysis in adipose tissue, and hepatic de novo lipogenesis. In hepatocytes, FFAs undergo acyl-CoA synthase activity and form fatty acyl-CoA, which can enter the β-oxidation cycle or undergo esterification [73,74]. However, in MASLD, inhibition of triglyceride incorporation in very low-density lipoprotein (VLDL) followed by decreased FFA oxidation occurs simultaneously with lipotoxicity and toxic metabolite generation and therefore leads to the worsening of liver damage, leading to steatohepatitis [75]. De novo lipogenesis in the liver is promoted by the activation of transcription factors such as sterol regulatory element-binding protein 1 (SREBP-1), carbohydrate response element-binding protein (ChREBP), and peroxisome proliferator-activated receptor (PPAR)-γ [76]. FFAs in hepatocytes induce alterations in insulin signaling pathways and contribute to insulin resistance. Lipotoxicity impairs insulin signaling by promoting oxidative stress and reactive oxygen species (ROS) generation and by stimulating inflammatory pathways, leading to steatosis progression to NASH, fibrosis, and cirrhosis [77,78]. It is known that lipotoxicity promotes cell death in MASLD and is called hepatocyte lipoapoptosis, whose degree correlates with the severity of MASLD [79,80]. When the lipid influx to the liver cannot be handled by mitochondrial or peroxisome function, respiratory oxidation processes may be altered and collapse with the impairment of lipid homeostasis and the increased generation of toxic lipid metabolites and ROS. Molecular oxygen, which accepts electrons, is the main source of radicals. The most important are hydroxyl radical (•OH), nitric oxide radical (NO•), and the superoxide anion (O_2_•−). These unstable and reactive radicals are generated as products of intracellular metabolic reactions and have the ability to react with proteins, free fatty acids, and DNA [81].

### 3.3. Lipid Accumulation in the Liver and Insulin Resistance

Insulin resistance and liver steatosis are bidirectionally connected, forming a vicious cycle, but their interplay still remains contradictory.

One of the proposed explanations is lipid droplet accumulation in hepatocytes. Diacylglycerols, ceramides, cholesterol esters, and saturated fatty acids are closely linked to the development of insulin resistance. In several animal studies, it has been shown that there is a correlation between the accumulation of lipid metabolites in the liver and the development of insulin resistance [82,83]. In mice lacking fatty acid transporter protein 5 (Fatp5), improvement of steatosis and systemic insulin sensitivity was evident [84]. Another animal study in leptin-deficient ob/ob mice showed that liver-specific inhibition of ChREBP improves hepatic steatosis and insulin resistance [85,86]. The alleviation of liver steatosis further led to decreased levels of plasma triglycerides and NEFAs, and finally, insulin sensitivity was restored in both skeletal muscles and adipose tissue. Since ChREBP is a major modulator of hepatic triglyceride concentration through the regulation of lipogenesis and triglyceride synthesis, this represents the pathway for worsening insulin resistance due to hepatic lipid accumulation [85]. Another proposed mechanism is the reduced activity of hepatic carnitine palmitoyl transferases (CPTs), which are important for long-chain fatty acid (LCFA) oxidation since they are capable of being transported through the mitochondrial membrane [87]. Db/db mice with increased expression of CPT1A and CPT1AM were protected against obesity-induced weight gain, hepatic steatosis, and insulin resistance. These animals also showed reduced serum glucose and insulin levels [88]. A study in mice with hepatic fat accumulation without increased peripheral adipose tissue showed that accumulated fat in the liver led to impaired insulin activation of AKT2 and inactivation of GSK3. Additionally, treatment with 2,4,-dinitrophenol, improved insulin signaling through mitochondrial uncoupling and promoted fat oxidation in the liver [89].

### 3.4. Hepatic Inflammation and Insulin Resistance

Steatosis is known to trigger and promote inflammation in the liver, and a lot of studies have developed animal models to examine the role of hepatic inflammation in insulin resistance pathogenesis. In a mouse model of MASLD induced by deficiency of methionine and choline, without obesity and peripheral fat accumulation, liver histology showed increased inflammatory infiltrates [90], followed by increased expression of proinflammatory cytokines IL-6 and TNF, while anti-inflammatory IL-10 expression was decreased [91,92]. One of the most important transcription regulators of proinflammatory cytokines in NASH is NF-κB [93]. Its activation in NASH is not only crucial for the persistence of an inflammatory state but also contributes to insulin resistance. NF-κB activation is regulated by IKK2. When IKK2 becomes activated, it phosphorylates IκBα, the inhibitor of NF-κB, which then becomes ubiquitinated and subsequently degraded. IKK2 is a serine–threonine kinase that is able to phosphorylate serine IRS and thus block signal transmission from insulin receptors into the cell’s cytoplasm [94]. This further releases NF-κB for translocation into the nucleus and promotes the transcription of proinflammatory genes. A recent study showed that a nitriles-rich fraction, such as the strong nuclear factor erythroid 2–related factor 2 (Nrf2) inducer and inhibitor of NF-κB, significantly reduced inflammation and improved insulin sensitivity and NASH histopathology [95]. Macrophages in the liver are considered to contribute to hepatic insulin resistance progression. Chronic excess calorie intake induces inflammation and ER stress in the liver. Furthermore, inflammatory and ER stress signaling pathways lead to insulin resistance progression through the inhibition of insulin signaling and the activation of the enzymes responsible for gluconeogenesis [35]. In a liver failure mouse model, ER stress induced the expression of proinflammatory cytokines and activated the NF-κB pathway [96]. Additionally, disturbances in lipid metabolism accompanied by gut-derived endotoxins promote the production and release of proinflammatory IL-1, IL-6, and TNF-α, which are able to inhibit insulin receptors signaling, aggravating insulin sensitivity and contributing to insulin resistance worsening [97,98]. In the presence of inflammatory factors such as interferon-gamma (IFN-γ), ligands for toll-like receptors (TLR), and cytokines, M2 liver macrophages undergo activation to M1, inducing the production of TNF-α and chemokines. Chemokines further activate leukocytes and stimulate their chemotaxis, contributing to inflammation and secondary insulin resistance [97,99]. Kupffer cells are proposed to play a significant role in hepatic inflammation [100]. They release cytokines and chemokines as a response to endogenous and exogenous molecular signals, further stimulating the recruitment of more macrophages or other cells of the immune system. As they are being activated, (M1) Kupffer cells inhibit insulin signaling in hepatocytes, probably mediated via TNF-α secretion [101].

### 3.5. Liver Oxidative Stress and Insulin Resistance

Oxidative injury and prolonged ROS overproduction are common features in fatty liver disease and play an important role in NASH progression. Increased lipid peroxidation and nitrosative stress in the liver have been confirmed in various models of MASLD [91,102,103]. The major characteristic of oxidative stress is excess endogenous ROS, which causes cell damage and alters signaling pathways. Superoxide anion, hydrogen peroxide, and hydroxyl radical ions are mostly generated in the mitochondria and peroxisomes. Oxidative stress parameters are found to correlate with neutrophil numbers and liver injury degree [104]. In obesity and MASLD, increased lipid accumulation represents an increased substrate for oxidation, resulting in increased mitochondrial production of O_2_^−^ and H_2_O_2_. In addition, ER stress and unfolded protein response with increased activation of NADPH oxidase contribute to more ROS generation [105]. The increase in ROS activates casein kinase-2 (CK2), which further activates the retromer complex for the degradation of GLUT4 [106,107]. A study by Matsuda et al. showed that insulin resistance can be prevented by restricting mitochondrial overactivation and the overproduction of ROS [108]. Oxidative stress contributes to insulin resistance directly as mentioned above, but also, increased ROS levels stimulate NF-κB, JNK, and p38 mitogen-activated protein kinase (MAPK), resulting in mitochondrial stress response and triggering inflammation which can further aggravate cell signaling and insulin resistance [109,110]. A study on diabetic rats showed that ellagic acid improved hepatic insulin sensitivity and lipid metabolism by reducing oxidative stress through Nrf2 and the hypoxia-inducible factor 1-alpha (HIF-α) pathway [111]. In addition, ROS can activate IKKβ, while IKKβ hepatic deficiency in mice fed a high-fat diet protected them from developing insulin resistance [112].

### 3.6. The Role of Hepatokines in Insulin Resistance

In MASLD, the secretion of hepatokines, hormone-like proteins, primarily by hepatocytes is disturbed and impairs signaling pathways, causing metabolic dysregulation in the liver. Hepatokines play an important role in communication and information transmission between the liver and target organs, such as adipose tissue and muscles [113].

Fetuin-A is a glycoprotein belonging to the cisplatin superfamily synthesized in hepatocytes and mainly serves as a transporter protein in the bloodstream. Apart from the liver, visceral and subcutaneous adipose tissue are additional sources of Fetuin-A [114]. Fetuin-A plays a key role in the pathogenesis of various clinical conditions such as insulin resistance [115], T2DM [116,117], MASLD [118], cardiovascular diseases [119], tumors, and nervous system disorders [120]. In patients with MASLD, obesity and insulin resistance serum levels of Fetuin-A are increased. In a mouse model of insulin resistance, after three days of high-fat diet feeding, the liver mRNA expression of Fetuin-A was significantly increased followed by liver steatosis, liver IR, and macrophage activation [115]. This hepatokine represents an important link between obesity and insulin resistance since it is known that Fetuin-A inhibits insulin signaling through the inhibition of IRS-1 phosphorylation in activated tyrosin kinase insulin receptors [120]. An additional mechanism is through the impairment of insulin-mediated glucose uptake by the decreased phosphorylation of Akt and AS160 downregulating GLUT4 translocation to the plasma membrane [121]. Furthermore, Fetuin-A was shown to stimulate inflammation through increased production of proinflammatory cytokines in monocytes and adipocytes and also through acting as an endogenous ligand for TLR4 [122]. It has been shown that Fetuin-A downregulates the production of adiponectin, affecting systemic insulin resistance [113]. On the other hand, knockout mice lacking Fetuin-A showed improved insulin signaling and prevented obesity development after being fed a high-fat diet [123].

Fetuin-B is similar to Fetuin-A and is also primarily produced in the liver. In vitro studies on cultured hepatocytes showed that Fetuin-B induced insulin resistance and stimulated lipid accumulation in cells’ cytoplasm by lowering phospho-AMPK levels and activating the liver-X-receptor (LXR)-SREBP1c pathway [124]. Liver-specific Fetuin-B knockout mice showed improved insulin sensitivity and glucose tolerance [125]. In women with polycystic ovary syndrome, an increased concentration of serum Fetuin-B was positively correlated with serum TNF-α, suggesting that Fetuin-B may potentially be related to low-grade inflammation [126]. A study on an obese population showed that leptin directly activated the transcription and expression of Fetuin-B in the liver in a STAT3-dependent manner [127]. In contrast to Fetuin-A which seems to predominantly modulate insulin signaling, Fetuin-B was found to affect glucose effectiveness without impairing insulin signaling [105,128,129].

Fibroblast growth factor 21 (FGF21) is predominantly released from the liver but can also be found in white and brown adipose tissue and the pancreas [130]. Recent studies have shown that circulating levels of FGF21 positively correlate with the severity of MASLD and the steatosis degree [131]. In obese/overweight people, increased levels of FGF21 were evident and were correlated with triglycerides, insulin concentration, and insulin resistance [132], suggesting that FGF21 may serve as a biomarker and early indicator of MASLD severity and progression. In diet-induced obese mice, treatment with FGF21 reduced the animals’ body weight and liver steatosis. The same study showed that FGF21 increases fatty acid and lipoprotein uptake, reduces lipogenesis, and increases VLDL secretion [133]. FGF21 improves insulin sensitivity in brown adipose tissue through induction uncoupling protein-1 (UCP-1) expression, thus lowering plasma glucose levels [134]. In brown adipose tissue, FGF21 stimulates thermogenesis, while in white adipose tissue, it stimulates adiponectin secretion and inhibits lipolysis [135]. In the liver, FGF21 has been shown to increase the expression of PPARγ coactivator-1α, improving mitochondrial function, which induces FFA oxidation, preventing its conversion into triglycerides and overaccumulation in hepatocytes [136]. Recent studies have pointed to the possibility of FGF21 preventing NASH progression into HCC through its anti-inflammatory effects through the inhibition of the hepatocyte-TLR4-IL17A signaling pathway [137]. In addition, FGF21 exerts pro-autophagic effects, stimulating lipid degradation. The administration of FGF21 to obese mice improves hepatic autophagy and steatosis via the Jumonji-D3 signaling pathway [138].

Selenoprotein P was identified as a carrier protein responsible for selenium transportation from the liver to other tissues such as the testes and brain [139]. An increase in selenoprotein P was found in humans with diabetes type 2, MASLD [140,141], and cardiovascular diseases [142]. In animal models of obesity, upregulation of hepatic selenoprotein P expression was evident, and it was negatively correlated with adiponectin concentration [143]. In a mouse model of diabetes type 2, the administration of antibodies against selenoprotein P improved glucose tolerance and insulin secretion [144].

## 4. Therapeutic Strategies

So far, the major therapeutic strategies in MASLD and obesity have been related to changes in dietary habits and reducing calorie intake, with physical activity suggested according to overall health [145]. These lifestyle changes still remain the cornerstone of MASLD therapy. However, in numerous cases, it is not always easy to obtain significant benefits and improvement only through lifestyle changes, so pharmaceutical therapy is required as an important support in the treatment of obesity, MASLD, and metabolic syndrome [146]. The most commonly used therapy is metformin, often in combination with statins and other lipid-lowering agents and antioxidant supplementation [147]. Targeting ER and UPR signaling in insulin resistance and T2DM might be a viable solution and therapeutic approach. It has been shown that AMPK activation inhibited ER stress-induced SREBP-1 and thus prevented the development of lipid-induced ER stress [44,148]. Another study using the plant sterol ester of α-linolenic acid improved MASLD by alleviating ER stress-triggered apoptosis through AMPK activation [149]. Lipid accumulation in hepatocytes can cause disturbances in Ca^2+^ metabolism by inhibiting sarcoplasmic/ER Ca^2+^ ATPase which induces ER stress and negatively affects glucose homeostasis in hepatocytes. Therefore, targeting Ca^2+^ channels to inhibit calcium efflux can contribute to enhancing protein folding capacity in ER [150]. A recent study suggests targeting mitochondrial dysfunction in NASH. Mitochondrial division inhibitor 1 (Mdivi1) was shown to restore mitochondrial homeostasis and function potentially through inhibition of the JNK/mitochondrial fission factor pathway [151]. Moreover, Mdivi1 exerts anti-inflammatory effects by reducing the mobilization of macrophages in injured hepatocytes, promoting the M1 phenotype. An in vitro study by Jin et al. identified a novel JNK inhibitor JM-2 that significantly reduced hepatocyte inflammation and apoptosis, while in mice fed with a high-fat diet JM-2, it protected the mice’s livers from lipid accumulation, inflammation fibrosis, and apoptosis [152]. Another interesting potential target might be the stimulation of mitophagy in hepatocytes. Yao et al. showed that blocking mitophagy suppression induced by a high-fat diet significantly alleviated the MASLD course and ameliorated mitochondrial dysfunction and lipid accumulation through the PINK1/Parkin pathway [153]. Similar research reported that quercetin improves MASLD via the promotion of AMPK-mediated hepatic mitophagy, suggesting that promoting mitophagy through AMPK upregulation could be a promising therapeutic strategy in MASLD treatment [154]. The modulation of IRE1 activity is suggested as a potential therapeutic target, since IRE1 kinase inhibitor application significantly attenuated nucleotide-binding oligomerization domain (NOD) 1 and NOD2-mediated proinflammatory response [45]. Novel therapeutic strategies include targeting clock genes and the resynchronization of disrupted biological clocks, which are involved in metabolic derangement [155,156]. Recent studies on suggested potential therapeutic agents are shown in the table, which can be further investigated for more beneficial effects and detailed mechanisms of action (Table 1).

## 5. Conclusions

Insulin resistance is defined as an inadequate response of target tissues to insulin stimulation, primarily the liver, adipose tissue, and muscles. Excess fat accumulation seems to be the major triggering factor for insulin resistance development. At the beginning, low-grade inflammation occurs in adipose tissue, which further contributes to insulin resistance development, firstly in adipose tissue and then in the liver, leading to MASLD development. MASLD tends to progress in NASH due to oxidative stress and inflammation that occur in the liver, which, together with lipotoxicity, aggravate insulin resistance, forming a vicious circle. Recent studies have pointed to hepatokines’ important role in communication between the liver and target organs, such as adipose tissue and muscles. Nowadays, ER stress is considered a very interesting contributor in the pathogenesis of metabolic disorders such as MetS and MASLD, and therefore, many therapeutic strategies are oriented toward targeting UPR components. In addition, pharmacological studies intend to modulate and recover biological clock and circadian dysrhythmia, which have been shown to significantly affect lipid and glucose metabolism. However, it is still necessary to investigate novel and more precise molecular mechanisms and signaling pathways that bidirectionally connect MASLD and insulin resistance.

## Figures and Tables

**Table 1:** Novel therapeutic agents with potential beneficial effects on insulin resistance and metabolic disorders.

Proposed Therapeutic Agents	Effects	References
Umbelliferone	Reduction in ER-stress-mediated apoptosis. Decrease in lipogenesis markers (SREBP1 and PPARγ)	[157]
*Andrographis paniculata* (Burm. f.) Nees	Modulation of the IRS-1/GLUT-2 pathway due to IL-6 inhibition	[158]
Icariin	Reduction in thioredoxin-interacting protein (TXNIP) and suppression of ER stress	[159]
Lunasin	Regulation of anti-inflammation, anti-oxidation, and glucose utilization and amelioration of glucose uptake	[160]
Glucosamine	Inhibition of the LPS/TLR4/NF-κB pathway	[161]
D-allulose	Anti-inflammatory effects through the suppression of INF-γ and the enhancement of macrophage function	[162]
Syzygium cumini	Partial agonism of PPARγ and an increase in the expression of adiponectin, an insulin sensitizer	[163]
Morin and 1-deoxynojirimycin	Suppression of cytokine signaling 3 and CD36/Serbp1/Fas signaling and promotion of PPARγ	[164]
Nobiletin	Suppression of adipocyte development in a clock-dependent manner	[155]

## Data Availability

Not applicable.
